# Ensemble Wavelet Decomposition-Based Detection of Mental States Using Electroencephalography Signals

**DOI:** 10.3390/s23187860

**Published:** 2023-09-13

**Authors:** Smith K. Khare, Varun Bajaj, Nikhil B. Gaikwad, G. R. Sinha

**Affiliations:** 1 Department of Electrical and Computer Engineering, Aarhus University, 8000 Aarhus, Denmark; 2 Indian Institute of Information Technology, Design and Manufacturing (IIITDM) Jabalpur, Jabalpur 482005, India; 3 International Institute of Information Technology, Bangalore 560100, India

**Keywords:** feature fusion, ensemble decomposition techniques, mental state recognition, optimized ensemble classifier, electroencephalogram signals

## Abstract

Technological advancements in healthcare, production, automobile, and aviation industries have shifted working styles from manual to automatic. This automation requires smart, intellectual, and safe machinery to develop an accurate and efficient brain–computer interface (BCI) system. However, developing such BCI systems requires effective processing and analysis of human physiology. Electroencephalography (EEG) is one such technique that provides a low-cost, portable, non-invasive, and safe solution for BCI systems. However, the non-stationary and nonlinear nature of EEG signals makes it difficult for experts to perform accurate subjective analyses. Hence, there is an urgent need for the development of automatic mental state detection. This paper presents the classification of three mental states using an ensemble of the tunable Q wavelet transform, the multilevel discrete wavelet transform, and the flexible analytic wavelet transform. Various features are extracted from the subbands of EEG signals during focused, unfocused, and drowsy states. Separate and fused features from ensemble decomposition are classified using an optimized ensemble classifier. Our analysis shows that the fusion of features results in a dimensionality reduction. The proposed model obtained the highest accuracies of 92.45% and 97.8% with ten-fold cross-validation and the iterative majority voting technique. The proposed method is suitable for real-time mental state detection to improve BCI systems.

## 1. Introduction

Recent technological developments have changed the roles of humans in safety-critical and complex areas, such as autonomous driving vehicles, aviation, healthcare systems, industries, etc., from manual to autonomous control systems [1]. However, due to the involvement of humans in several tasks, at the same time, the growing sophistication of these processes makes human intervention and control difficult. Therefore, there is an urgent need for the development of more accurate and automated systems. An analysis of an individuals’ cognitive, emotional, and psychological states can provide a solution using brain–computer interface (BCI) technologies [2]. Such information measures the mental states of the users to make these environments safer for human–machine interfaces. The brain’s physiological activities have been studied by electroencephalograms (EEGs) [3], functional magnetic resonance imaging (fMRI) [4], functional near-infrared spectroscopy (fNIRS) [5], magnetoencephalograms (MEGs) [6], and other forms of biosignals, such as electrooculograms (EOGs) [7], electrocardiograms (ECGs) [7,8], and galvanic skin responses (GSRs) [9], to detect various conditions [10,11]. Taking the day-to-day perspective of a mental activity measurement, issues related to size, weight, expense, power consumption, and radioactivity restrict the usage of MEGs and fMRI [12]. EOG, ECG, and GSR signals provide some degree of correlation with mental states (mental fatigue, drowsiness, and stress) [10]. However, such techniques have demonstrated success only in combination with neuro-imaging methods linked to the central nervous system [10]. As a result, fNIRS and EEG signals proved the most appropriate choices for BCI systems [10]. EEG signals are favored over fNIRS signals, as they offer higher sensitivity to variations in brain activities and higher temporal resolution [10]. Moreover, researchers have widely used EEG signals to study emotions, cognitive load, fear of missing out, drowsiness, and schizophrenia, due to their low-cost, portable, and non-invasive properties [13,14,15,16,17,18].

Recently, many studies have been presented for detecting mental states using EEG signals. The mental states of “workload”, “fatigue”, and “situational awareness” have been studied by examining the correlation between mental workload and EEG signals in different conditions, such as in airplane pilots and car drivers [11]. Myrden et al. presented an EEG-BCI model to predict the mental states of frustration, fatigue, and attention. Different features extracted using the fast Fourier transform (FFT) have been classified with linear discriminant analysis (LDA), support vector machines (SVMs), and naive Bayes classifiers [19]. Li et al. recognized reading silently, a comprehension task, a mental arithmetic task, and a question-answering task based on the self-assessment Manikin (SAM) model [20]. Nuamah et al. classified five tasks (baseline, visual counting, geometric figure rotation, letter composition, and multiplication) using the short-time Fourier transform (STFT) to extract different features, which were classified using an SVM classifier [21]. Liu et al. presented a frequency domain analysis of features using the FFT in combination with SVM to detect attentive and inattentive mental states of students [22]. Ket et al. classified attention, no attention, and rest states using sample entropy and linear features with an SVM classifier [23].

Wang et al. used the focus of attention ability during mathematical problem solving and lane-keeping driving tasks. The central, parietal, frontal, occipital, right-motor, and left-motor power spectra computed using filtering and independent component analysis (ICA) were classified with an SVM classifier [24]. Djamal et al. evaluated features from raw EEG signals and wavelet decomposition to recognize attention and inattention activities [25]. Arico et al. used stepwise linear discriminant analysis and the statistical test of analysis of variance (ANOVA) to detect easy, medium, and hard mental assessments [12]. Hamadicharef et al. developed an attention and non-attention classification state model using a combination of filter banks, common spatial patterns, and a Fisher linear discriminant classifier [26]. Mardi et al. used Log energy, Higuchi, and Petrosian’s fractal dimension to extract chaotic features for detecting alertness and drowsiness states [27]. Richer et al. evaluated the band power of frequency bands. They computed histograms of naive and entropy-based scores using the P2 algorithm and classified them with binary classifiers [28]. Aci et al. used STFT-based features to detect focused (F), unfocused (UF), and drowsiness (D) mental states [29]. Zhang et al. used six convolutional networks and one output layered deep neural network to predict F, UF, and D states [30]. Islam et al. explored multivariate empirical mode decomposition (MEMD) and the discrete wavelet transform (DWT) to detect working and relaxed states. The nonlinear features extracted from intrinsic mode functions and subbands (SBs) have been classified with an ensemble classifier [31]. Tiwari et al. used rhythm level analysis using filtering and the FFT. The SVM, k-nearest neighbor (KNN), and random forest classifiers have been used to detect high- and low-level attention [32]. Samima and Sarma used an analysis of rhythms using filtering and artificial neural network (ANN) classifiers for mental workload level assessments [33]. Mohdiwale et al. used a DWT-based rhythm analysis using teaching–learning-based optimization for detecting cognitive work assessments [34]. Easttom and Alsmadi presented a comparative analysis of EMD and variational mode decomposition to extract nonlinear entropy and Higuchi features for mental state detection [35]. Khare et al. used wavelet-based analysis using only the rational dilation wavelet transform (RDWT) to extract five statistical and nonlinear features and classified them using an ensemble classifier to detect various mental states [36]. Kumar et al. used analysis of EEG rhythms using the discrete Fourier transform and power spectral density (PSD) to detect mental states using the KNN classifier [37]. Rastogi and Bhateja explored artifacts of or noise elimination in mental state EEG signals using a stationary wavelet transform (SWT)-enhanced fixed-point fast ICA technique [38].

The methods in the literature used traditional feature extraction from raw EEG signals, statistical analysis, filtering techniques, frequency-based transforms such as the FFT or STFT, rhythm-based analysis, and wavelet-based decomposition. However, direct feature extraction exhibits a decreased performance [15], frequency-based transforms result in a time–frequency trade-off [15], filtering and rhythmic analyses require choosing filter coefficients [15], and wavelet-based methods require the selection of a mother wavelet [15]. The experimental and empirical selection of parameters can cause information loss and performance degradation due to misclassification [15]. Thus, to overcome these shortcomings, we propose an ensemble-based analysis using advanced decomposition techniques, including the tunable Q wavelet transform (TQWT), the multilevel DWT (MDWT), and the flexible analytic wavelet transform (FAWT). Individual and feature fusion for the automated detection of three mental states (F, UF, and D) is accomplished with an optimizable ensemble technique. The major contributions of the proposed work are listed below:

- Analysis of ensemble decomposition techniques using multi-wavelet decomposition.
- Statistical analysis to reduce the feature dimensions of multi-wavelet feature analysis for mental state detection.
- Analysis of feature fusion to detect the best combination of features.
- Exploring an optimized ensemble classifier to determine the optimum hyper-parameter selection.

The remainder of paper is organized as follows: Section 2 explains the methodology. The results are presented in Section 3. The discussion and conclusions are presented in Section 4 and Section 5.

## 2. Methodology

**Figure 1:**
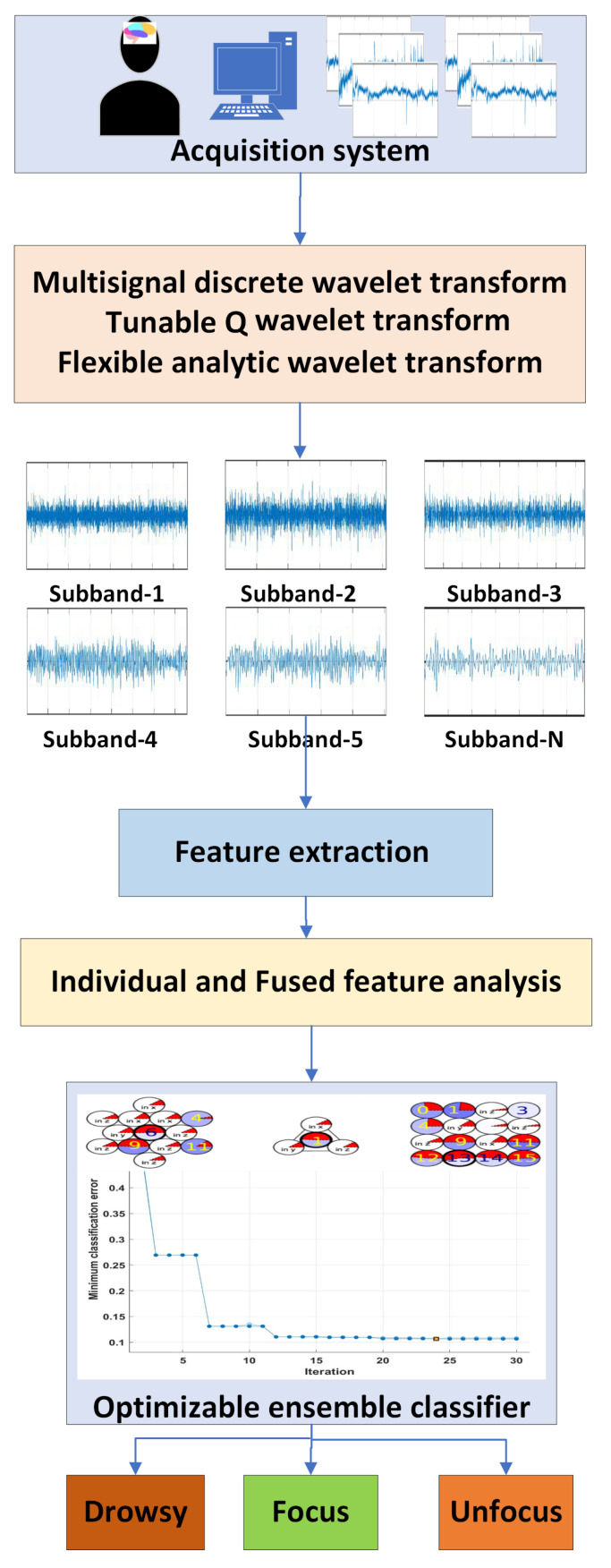
Proposed ensemble model for mental state detection.

The proposed methodology comprises several steps, such as EEG dataset pre-processing, signal analysis using ensemble decomposition, feature extraction, and classification. The flowchart of the method is shown in Figure 1.

### 2.1. Dataset and Preprocessing

The EEG signals of mental states from Kaggle were used, which is a public dataset repository [29,39]. The EEG recordings from five subjects originally consisted of a total of 25 h of recording. The participants performed train control on the “Amtrak-Philadelphia” route using the Acela-express simulator. The subjects were instructed to maintain the locomotive speed at 40 mph in every experiment. Each subject controlled the train for 35 to 55 min. The subjects performed seven experiments each, performing at most one experiment per day. The focused state was captured by paying attention to simulator control during the first 10 min of the experiment. The participants became unfocused and stopped paying attention during the second 10 min, exhibiting an unfocused state. Finally, the participants closed their eyes, relaxed freely, and dozed off during the next 10 min to capture the drowsy state. The recording of EEG data was in accordance with international 10–20 standards using an EPOC EEG system. A voltage resolution of 0.51 μV, a sampling frequency of 128 Hz, and a bandwidth between 0.2 and 43 Hz were chosen for data acquisition and pre-processing. A 10 min segment of each class was stratified into 30 s non-overlapping EEG segments with 3840 samples. Each class consists of a total of 680 EEG segments. The dataset details are available in [29,30,39].

### 2.2. Ensemble Decomposition Techniques

In this paper, we have explored an ensemble of three wavelet-based analyses. A brief description of MDWT, TQWT, and FAWT is given in the following subsections.

#### 2.2.1. Multilevel Discrete Wavelet Transform (MDWT)

The MDWT decomposes the signal into two bands called low-pass (LP) and high-pass (HP) filter banks, respectively. The LP filter bank captures the low-frequency content of the signal, while the HP filter bank captures the high-frequency content of the signal. Decomposition of EEGs into four levels results in four HP SBs and one LP SB. The mathematical formulation of the MDWT for the *j*th level of decomposition is defined as [40]

(1) $$\begin{array}{c} V_{\phi }^{\left(j\right)}\left(k\right)=\sum_{i=1}^{M-j+1}\phi \left(i-2k\right)V_{\phi }^{\left(j-1\right)}\left(i\right),\\ V_{\theta }^{\left(j\right)}\left(k\right)=\sum_{i=1}^{M-j+1}\theta \left(i-2k\right)V_{\phi }^{\left(j-1\right)}\left(i\right),\\ k=1,2,\ldots ..,2^{n-i}, \end{array}$$

where *M* is the length of the signal ($M=2^{n}$), ϕ and θ are LP and HP filter, $V_{\phi }$ is the LP-filtered signal, and $V_{\theta }$ is the HP-filtered signal.

#### 2.2.2. Tunable Q Wavelet Transform (TQWT)

The traditional forms of wavelet transforms decompose any signal into subsequent LP SBs and HP SBs with a choice of the mother wavelet. Accurately choosing a wavelet to extract meaningful information is another topic of discussion. The TQWT does not require the selection of a mother wavelet. The decomposition into LPSBs and HPSBs using the TQWT requires tuning parameters, namely the quality factor (*q*), the oversampling rate (*R*), and decomposition levels (*B*), respectively [41]. The quality factor (*q*) is chosen as 1 for non-oscillatory signals, and it is >1 for oscillatory signals [41]. *R* controls the localization of the time-domain response, and it is selected as ≥3 to better capture the time-domain response [41]. The EEG signal can split into a number *B* of high-pass subbands (HPSBs) and one low-pass subband (LPSB) using B decomposition levels. The HPSBs and LPSBs are generated by filter-bank analysis with an LP and a HP frequency response of $U_{0}^{B}\left(\omega \right)$ and $U_{1}^{B}\left(\omega \right)$ denoted as [41]:

(2) $$U_{0}^{B}\left(\omega \right)=\left\{\begin{array}{cc} \prod_{b=0}^{B-1}U_{0}\left(\frac{\omega }{a^{b}}\right), & \left|\omega \right|\leq a^{B}\pi ,\\ 0, & a^{B}\pi <\left|\omega \right|\leq \pi , \end{array}\right.$$

(3) $$U_{1}^{B}\left(\omega \right)=\left\{\begin{array}{cc} U_{1}\left(\frac{\omega }{a^{B-1}}\right)\prod_{b=0}^{B-2}U_{0}\left(\frac{\omega }{a^{b}}\right), & \left(1-\beta \right)a^{B-1}\pi \\ \; & \leq \left|\omega \right|\leq a^{B-1}\pi ,\\ 0, & \omega \in [-\pi ,\pi ]. \end{array}\right.$$

The low-frequency and high-frequency components from any signal can be obtained by LP scaling (a) and HP scaling (β) denoted as [41]

(4) $$\beta =\frac{2}{q+1},$$

(5) $$a=1-\frac{\beta }{R}.$$

The quality factor is represented as [41]

(6) $$q=\frac{2-\beta }{\beta }.$$

The oversampling rate is denoted as [41]

(7) $$R=\frac{\beta }{1-a}.$$

#### 2.2.3. Flexible Analytic Wavelet Transform (FAWT)

The FAWT offers several benefits over the conventional dyadic wavelet transform, which includes a provision for arbitrary sample rates for LP and HP channels that allow flexible time-frequency covering. The HP channel used by the FAWT uses a complex pair of atoms, giving it more freedom in choosing the transform parameters. These advantages allow the FAWT to analyze complex oscillating signals, such as vibrations and EEG signals [42,43]. The iterative filter bank structure of the FAWT decomposes the signals into two HP channels and one LP channel, respectively [42]. The frequency responses of the LP and HP filter, denoted as $V_{\phi }\left(\omega \right)$ and $V_{\theta }\left(\omega \right)$, are defined as [42]

(8) $$\begin{array}{c} V_{\phi }\left(\omega \right)=\left\{\begin{array}{cc} \sqrt{\alpha_{1}\alpha_{2}}, & \mathrm{for}|\omega |<\omega_{p},\\ \sqrt{\alpha_{1}\alpha_{2}}\theta \left(\frac{\omega -\omega_{p}}{\omega_{s}-\omega_{p}}\right), & \mathrm{for}\;\omega_{p}\leq \left|\omega \right|\leq \omega_{s},\\ \sqrt{\alpha_{1}\alpha_{2}}\theta \left(\frac{\pi -\omega +\omega_{p}}{\omega_{s}-\omega_{p}}\right), & \mathrm{for}\;-\omega_{s}\leq \left|\omega \right|\leq -\omega_{p},\\ 0, & \mathrm{for}\;|\omega |\geq \omega_{s}, \end{array}\right.\\ V_{\theta }\left(\omega \right)=\left\{\begin{array}{cc} \sqrt{2\alpha_{3}\alpha_{4}}\theta \left(\frac{\pi -\omega -\omega_{0}}{\omega_{1}-\omega_{0}}\right), & \mathrm{for}\;\omega_{0}\leq \omega <\omega_{1},\\ \sqrt{2\alpha_{3}\alpha_{4}}, & \mathrm{for}\;\omega_{1}\leq \omega <\omega_{2},\\ \sqrt{2\alpha_{3}\alpha_{4}}\theta \left(\frac{\omega -\omega_{2}}{\omega_{3}-\omega_{2}}\right), & \mathrm{for}\;\omega_{2}\leq \left|\omega \right|\leq \omega_{3},\\ 0, & \mathrm{for}\;\omega \in \left[0,\omega_{0}\right)\cup \left(\omega_{3},2\pi \right), \end{array}\right. \end{array}$$

(9) $$\begin{array}{c} \omega_{p}=\frac{(1-\beta )\pi +\epsilon }{\alpha_{1}},\\ \omega_{s}=\frac{\pi }{\alpha_{2}},\\ \omega_{0}=\frac{(1-\beta )\pi +\epsilon }{\alpha_{3}},\\ \omega_{1}=\frac{\alpha_{1}\pi }{\alpha_{2}\alpha_{3}},\\ \omega_{2}=\frac{\pi -\epsilon }{\alpha_{3}},\\ \omega_{3}=\frac{\pi +\epsilon }{\alpha_{3}}, \end{array}$$

where $\alpha_{1}$ and $\alpha_{2}$ are the up-sampling and down-sampling factors of the LP channel, $\alpha_{3}$ and $\alpha_{4}$ are the up-sampling and down-sampling factors of the HP channel, $\omega_{p}$ is the pass-band frequency, and $\omega_{s}$ is the stop-band frequency. β and ϵ are factors related to perfect reconstruction.

**Figure 2:**
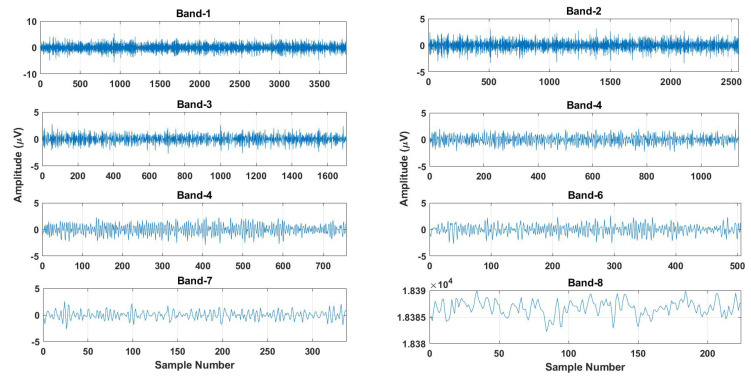
A typical example of SBs generated by the TQWT.

A typical example of the SBs obtained after seven levels of decomposition is represented in Figure 2.

### 2.3. Features Extraction

Features are crucial for drawing a decision boundary to improve system performance. Nonlinear, fractal dimension, and statistical features provide representative information for different physiological and neurological conditions [44,45]. Such features provide an effective representation of brain dynamics, which helps to improve the system performance [44,45]. The current work explores the application of 27 statistical and nonlinear features to detect three mental states. These features are the standard deviation, Hurst exponent, average energy, wavelength, V order, skewness, kurtosis, Hjorth mobility, Higuchi fractal dimension, Lyapunov exponent, differential absolute standard deviation value, absolute value of the summation of an exponential root, absolute value of the sum of square root, normalized first difference, normalized second difference, mean value of the square root, difference variance value, log energy, absolute energy, simple square integral, slope sign change, peak amplitude, minima, peak amplitude, zero crossing rate, interquartile range, and trimean [46,47,48,49,50].

### 2.4. Ensemble Classifiers

**Figure 3:**
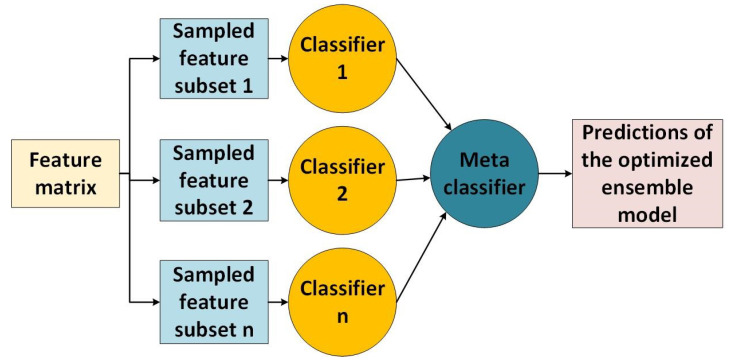
Typical working of ensemble classifier techniques.

Bootstrap aggregating is an ensemble method usually used to improve classification performance. This work combines five ensemble models to obtain the best optimum combination of hyper-parameters for classification. The classification techniques are ensemble bagged tree, ensemble boosted tree, random under-sampling boosted tree, ensemble subspace knn, and ensemble discriminant trees classifiers with hyper-parameters. In ensemble operation, bootstrap resampling is applied to divide the training data into subsets. Each subset is then used to construct a decision tree, and the output is a function of the voting scheme from the different sets of decision trees. The best-performing classifier is selected as a meta-classifier. Figure 3 shows the operations of ensemble classification techniques. In addition, the performance of classifiers is highly hyper-parameter dependent [51]. Careful selection of the hyper-parameters prevents the model from over-fitting and performance degradation. An accurate choice of hyper-parameters is time-consuming and prone to human error. To overcome this, we have explored an optimizable ensemble classification design using the Matlab classifier application.

The classification setting for a datum with pair $U_{i},V_{i}$, where (*i* = *1, 2, …, M*), $U_{i}$ is the predictor with a dimension *j*, and $V_{i}$ is the response with *K* numbers of classes, is described. The estimator function for classification is represented by [52]

(10) $$f\left(.\right)=h_{M}\left(\left(U_{1},V_{1}\right),\left(U_{2},V_{2}\right),\ldots ..\left(U_{M},V_{M}\right)\right),$$

where $h_{M}\left(.\right)$ is the estimator as a function of the input data. The ensemble algorithm is as follows [52]:

1. Construct $\left(U_{1}^{*},V_{1}^{*}\right),\left(U_{2}^{*},V_{2}^{*}\right),\ldots ..\left(U_{M}^{*},V_{M}^{*}\right)$ bootstrap samples *M* times randomly $\left(U_{1},V_{1}\right),\left(U_{2},V_{2}\right),\ldots ..\left(U_{M},V_{M}\right)$.
2. Evaluate the bootstrap estimator $f^{*}\left(.\right)=\left(U_{1}^{*},V_{1}^{*}\right),\left(U_{2}^{*},V_{2}^{*}\right),\ldots ..\left(U_{M}^{*},V_{M}^{*}\right)$.
3. Repeat steps 1 and 2 *L* times, where *L* = 50 or 100.
4. $f^{*l}\left(.\right)\left(l=1,2,\ldots ,L\right)$. Finally, the ensemble estimator is obtained as $f_{ensemble}\left(.\right)=L^{-1}\sum_{l=1}^{L}f^{*l}\left(.\right)$

### 2.5. Performance Measure

The evaluation of model performance is a crucial stage to measure the effectiveness of the developed model [53]. We have performed a comprehensive analysis of the developed ensemble model to test the effectiveness of the developed system. The evaluation strategy uses three stages. In the first stage, a model is evaluated for its consistency using different validation techniques, namely holdout cross-validation (HOCV), five-fold cross-validation (FFCV), and ten-fold cross-validation (TFCV) techniques. In HOCV, we used the 80:20 strategy, where training and testing were performed on 80% and 20% of the total data, respectively. In five- and ten-fold validation techniques, data were divided into five and ten equal parts, respectively. The model was trained and tested five and ten times, with one part used for testing and the remaining for training, respectively. In the second stage, we performed feature fusion and selected the most prominent features. Finally, we evaluated different performance measures to obtain insights into the developed model. Five evaluation matrices, accuracy, recall, specificity (*SPE*), precision (*PPV*), and F-1 score, were used to test the system performance. It is noteworthy to mention that we have used subject-independent training and testing to evaluate the model performance. The mathematical formulations of the performance parameters are expressed as follows.

(11) $$\begin{array}{c} Accuracy=\frac{T_{p}+T_{n}}{T_{p}+F_{p}+T_{n}+F_{n}},\\ Recall=\frac{T_{n}}{F_{p}+T_{n}},\\ SPE=\frac{T_{p}}{F_{n}+T_{p}},\\ PPV=\frac{T_{p}}{F_{p}+T_{p}},\\ F-1\;\mathrm{score}=\frac{2\times Recall\times PPV}{Recall+PPV}, \end{array}$$

where $T_{p},T_{n},F_{p},$ and $F_{n}$ are the values of true positive, true negative, false positive, and false negative, respectively.

## 3. Results

We aimed at classifying mental states using ensemble decomposition and classification algorithms. At first, stratification of the EEG signals was performed to obtain 3840 non-overlapping samples for each class. The stratified signals were decomposed into SBs using three wavelet-based decomposition techniques (MDWT, TQWT, and FAWT). We used four-level decomposition using Daubechies wavelet (db2), yielding five SBs corresponding to five EEG rhythms. The tuning parameters of the TQWT were chosen as q=2,R=5, and B=7. For the FAWT, the tuning parameters were selected as B=6,p=3,q=5,r=2, and s=3, respectively. We extracted 27 features from the SBs of the MDWT, FAWT, and TQWT with an empirical setting of the tuning parameters. The current analysis includes a feature matrix of all the channels with 27 features. Therefore, a total of 378 features with a total of 2040 segments were introduced into the ensemble classification techniques. The model uses three validation strategies, i.e., HOCV, FFCV, and TFCV. It is noteworthy to mention that we have maintained the same experimental setup. Table 1 shows the accuracy obtained for each SB using MDWT features. The accuracy of two-class and multiclass classification is highest for SB-1. The model yielded the highest accuracies of 95.07%, 94.93%, and 94.36% for D vs. F using HOCV, FFCV, and TFCV, respectively. For UF vs. F, the highest accuracies were 91.18%, 89.34%, and 88.60%, while for D vs. UF, the accuracies were 88.84%, 89.78%, and 88.53% using the optimizable ensemble classifier with HOCV, FFCV, and TFCV techniques. Similarly, three-class classification yielded the highest accuracies of 87.45%, 87.45%, and 86.27% using HOCV, FFCV, and TFCV.

The accuracy obtained for the TQWT features using an optimized ensemble classifier is shown in Table 2. The accuracy of SB-1 was higher then other SBs. For D vs. F, the optimizable model obtained the highest accuracies of 95.22%, 96.10%, and 94.85% with HOCV, FFCV, and TFCV. HOCV, FFCV, and TFCV for UF vs. F classification yield the highest accuracies of 93.01%, 91.32%, and 90.74%. For D vs. UF, the optimizable ensemble classifier yielded accuracies of 90.74%, 90.74%, and 90.22% with HOCV, FFCV, and TFCV. Similarly, HOCV, FFCV, and TFCV techniques yielded accuracies of 85.78%, 89.82%, and 89.02% for three-class classification.

Table 3 shows the accuracy obtained in each SB using FAWT-based features and the optimizable ensemble classifier. The analysis reveals that the last SB yielded the highest accuracy for different classification scenarios. Table 3 shows that the ensemble-based classifier yielded the highest accuracies of 97.79%, 96.91%, and 96.84% for D vs. F classification using HOCV, FFCV, and TFCV techniques. The model provided the highest accuracies of 93.75%, 92.28%, and 91.01% for UF vs. F, D vs. UF, and D vs. F vs. UF using the HOCV technique. The highest accuracies of 93.09%, 91.10%, and 90.90% for UF vs. F, D vs. UF, and D vs. F vs. UF were obtained with FFCV. The accuracies obtained with TFCV for UF vs. F, D vs. UF, and D vs. F vs. UF were 92.94%, 90.96%, and 90.10%, respectively.

**Figure 4:**
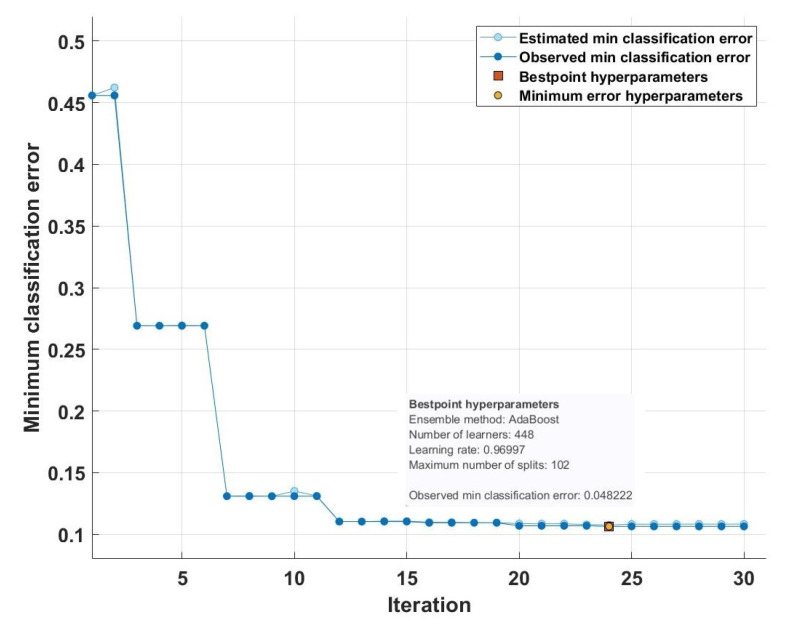
Training curve obtained for our optimizable ensemble classifier.

Thus, it is clear from Table 1, Table 2 and Table 3 that the accuracy of our developed model is almost stable for three validation techniques in various SBs for different classification scenarios. SB-1 generated the highest accuracy for MDWT and TQWT feature classification. The accuracy yielded by FAWT-based features was highest in SB-7. Analysis also reveals that FAWT-based features provide discernable characteristics, and due to this it obtained the highest accuracy over TQWT- and MDWT-based features. Further, our developed model is consistent for different classification scenarios (binary and multiclass analysis) with three validation techniques. The features provided by drowsy and focused classes are highly discernable; therefore, they yielded the highest classification rate over other scenarios. On the other hand, the features of focused and unfocused classes significantly overlap, resulting in a decreased model performance. An exemplary training curve obtained for the optimized ensemble classifier is shown in Figure 4.

**Figure 5:**
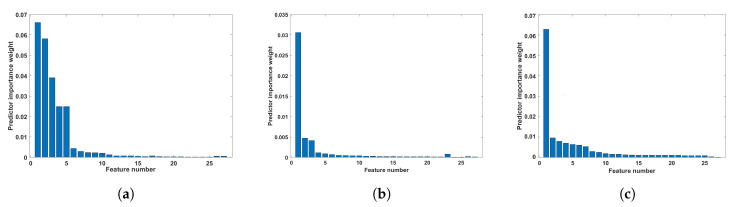
The feature rank obtained using the minimum redundancy feature selection technique: (**a**) FAWT, (**b**) TQWT, and (**c**) MDWT decomposition.

As stated earlier, our training and testing feature set comprised all features from all channels. Analysis of the model with all features may increase the time without improving the classification performance [54]. Therefore, we used feature ranking analysis to test our model performance with optimal features using the minimum redundancy feature selection technique. Figure 5 shows the feature rank obtained for FAWT, TQWT, and MDWT-based features. As seen from Figure 5, out of twenty-seven features, only a few features are statistically significant for classification. The feature importance values for FAWT, TQWT, and MDWT decrease significantly or remains the same after six features. This reveals that a similar performance can be obtained using less features with higher feature ranks.

To obtain an insight into our developed model, we explored a fusion of the most important features of the three decomposition techniques. During fusion, we concatenated the features from all channels according to their ranks. As evident from Table 1, Table 2 and Table 3, SB-1 for TQWT and MDWT and SB-7 for FAWT features yielded the highest accuracy. Therefore, we have fused the features from these SBs. Table 4 represents the accuracy obtained by feature fusion of decomposition techniques with different feature combinations. As seen from Table 4, the accuracy yielded by the ensemble model increases with an increase in the feature count. The model provides the highest performance with four features. After that, the accuracy of the model decreases slightly or remains constant. Furthermore, our model exhibits that feature fusion helps to improve system performance. The fusion of three decomposition techniques yielded the highest accuracy, followed by the features based on a fusion of TQWT and FAWT decomposition. The combination of TQWT and MDWT feature fusion resulted in the lowest performance. Further, to obtain the highest score, we evaluated the highest performance measures of TFCV using iterative majority voting (IMV). For IMV, we conducted multiple rounds of TFCV, and selected the one with the best overall and fold-wise accuracy. The model exhibited the highest accuracy of 97.8%, obtained twice during fold-wise analysis.

Further, we tested the model performance using four performance metrics, as shown in Table 5. The performance measures show that the drowsy class generated the most discriminant features with the highest recall, SPE, PPV, and F1 score. The focused class is the second best, while the worst performance is exhibited by the unfocused class. The analysis shows that feature fusion of the drowsy class yields the highest recall, SPE, PPV, and F1 score of 93.13%, 95.91%, 91.76%, and 92.44%. The recall, SPE, PPV, and F1 score yielded for the drowsy class was 97.12%, 99.63%, 99.26%, and 98.18% using the IMV technique.

**Figure 6:**
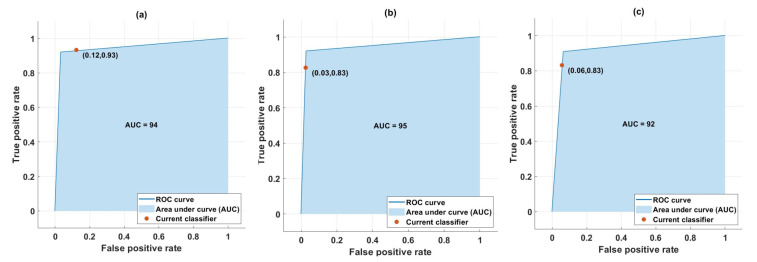
ROC and AUC obtained using fused features for (**a**) drowsy, (**b**) focused, and (**c**) unfocused classes.

To obtain more insight into the proposed system, the receiver operating characteristics (ROC) and area under the curve (AUC) were evaluated, as shown in Figure 6. The ROC and AUC of D vs. F and UF, F vs. D and UF, and UF vs. D and F states for fused features are shown in Figure 6a–c. It is evident that the AUC for drowsy is 94%, while for focused and unfocused states it is 95% and 92%, with an average accuracy of 93.67%, respectively.

## 4. Discussion

We have tested the efficacy of our proposed model by comparing it with existing state-of-the-art techniques. Borghini et al. [11] computed the power of alpha, theta, and delta frequency bands. An analysis of these frequency bands was performed, and they reported an accuracy of around 90%. Myrden et al. [19] used the FFT to evaluate frequency domain features and classified them with SVM, LDA, and naive Bayes classifiers. Their model yielded the highest accuracies of 71.6%, 74.8%, and 84.8% for frustration, fatigue, and attention levels using the LDA classifier. In another method by Liu et al. [22], an FFT- and SVM-based model yielded an accuracy of 76.82%. Li et al. [20] used an SAM model and obtained and average accuracy rate of 57.03% with the KNN classifier. Nuamah et al. [21] presented a combination of STFT and SVM for feature extraction and classification. Their method obtained an accuracy of 93.33% using the radial basis function kernel. Ket et al. [23] automatically identified three tasks, namely attention, no attention, and rest, using two experiments (ball playing or walking cartoon). The sample entropy and linear features classified using SVM and their method yielded an accuracy of 76.19% and 85.24% using two experiments with sample entropy features. Wang et al. [24] fed features extracted by filtering and ICA into an SVM classifier and achieved 86.2% and 84.6% accuracies in the classification of driving tasks and math-related activities. Djamal et al. [25] computed non-wavelet- and wavelet-based features and classified them with an SVM classifier, and their method provided accuracies in the range of 44–58% and 69–83%, respectively. Hamadicharef et al. [26] developed a filters, common spatial patterns, and Fisher linear discriminant-based attention and non-attention classification model with an accuracy of 89.4%. Chaotic features based on log energy, Higuchi, and Petrosian’s fractal dimension artificial neural network classifiers claim an accuracy of 83.3%. Richer et al. [28] used the power of frequency bands, naive, entropy scores, and a binary classification model to obtain a sensitivity of 82% and 80.4% and a specificity of 82.8% and 80.8% for the focus and relax scores, respectively. The methods discussed above have been tested on different datasets for mental state classification. The proposed method was compared with the work of Aci et al. [29] and Zhang et al. [30] on the same dataset, as shown in Table 6. Aci et al. used STFT-based feature extraction to compute different feature sets. ANFIS, SVM, and KNN classifiers were employed to classify 154 features with accuracies of 81.55%, 77.76%, and 91.72%, respectively. A method by Zhang et al. [30] used a deep-learning-based convolutional neural network (CNN) and provided an accuracy of 96.4%. Kumar et al. explored the analysis of PSD using FFT-based feature extraction and a KNN classifier. The channel-wise and grouped channel analysis yielded accuracies of 80% and 97.5% [37]. Khare et al. used RDWT wavelet analysis with statistical feature extraction. The classification of features resulted in an accuracy of 91.77% using the bagged tree classifier [36]. Rastogi and Bhateja et al. [38] performed elimination of artifacts and noise using SWT and ICA. However, their group did not report the classification accuracy. In our method, we have used ensemble-based decomposition and extraction of nonlinear features. The individual analysis of MDWT, TQWT, and FAWT features yielded accuracies of 88.27%, 89.02%, and 90.1%. Fused feature analysis yielded accuracies of 90.98%, 88.62%, and 89.61% for TQWT/FAWT, TQWT/MDWT, and MDWT/FAWT feature fusion using the TFCV technique. A combined fused feature analysis using TFCV and IMV resulted in accuracies of 92.45% and 97.8%. The analysis shows that our developed model has surpassed the performance of existing state-of-the-art techniques, showing the efficacy of our developed model.

## 5. Conclusions

The proposed method classifies focused, unfocused, and drowsy mental states. We have developed an ensemble decomposition and optimized classification technique to create an effective model for mental state detection. Our analysis shows that feature extraction using the FAWT yields the most discernible features for detecting mental states. We demonstrated that feature fusion is helpful for the extraction of representative SBs from EEG signals. Hence, it is useful for extracting meaningful information for mental state analysis. Our model also shows that feature fusion with statistical analysis helps to reduce the feature dimensions with an increased accuracy. The model yielded an accuracy of 97.8% with IMV, which is higher than existing state-of-the-art techniques. Our developed model can detect drowsy, focused, and unfocused states with accuracies of 99.26%, 98.52%, and 94.11%. The proposed work is ready for real-time mental state classification applications to take brain–computer and human–machine interfaces to the next level. The advantages of our developed model are listed as follows:

- The model can explore multi-level ensemble wavelet analysis.
- The model is effective and robust due to comprehensive analysis.
- The optimized ensemble classifier allows tuning of the hyper-parameters to achieve the best classification performance.
- The model yielded the highest accuracy of 97.8%.
- The model supports binary and multi-class analyses.

The model has following limitations:

- The model has been tested on a single EEG dataset.
- The dataset contains fewer subjects.
- The model has not been tested with leave-one-subject-out classification.

In the future, we will aim to:

- Perform adaptive parameter tuning and channel selection.
- Develop model leave-one-subject-out classification on a relatively larger dataset.

## Figures and Tables

**Table 1:** The overall accuracy (%) obtained in MDWT SBs for HOCV, FFCV, and TFCV techniques using different classification scenarios.

Class	SB-1	SB-2	SB-3	SB-4	SB-5
HOCV					
D vs. F	95.07	94.12	93.53	95.59	88.97
UF vs. F	91.18	88.97	90.07	88.60	81.62
D vs. UF	88.84	86.40	87.13	88.60	80.51
D vs. UF vs. F	87.45	84.61	84.12	81.47	75.98
FFCV					
D vs. F	94.93	93.53	92.21	93.09	89.19
UF vs. F	89.34	88.97	88.16	85.15	82.65
D vs. UF	89.78	88.60	87.06	86.76	81.10
D vs. UF vs. F	87.45	84.61	84.12	81.47	75.98
TFCV					
D vs. F	94.26	93.82	92.79	92.94	88.97
UF vs. F	88.60	88.24	88.24	86.69	81.40
D vs. UF	88.53	87.65	88.01	85.51	79.12
D vs. UF vs. F	86.27	83.33	81.57	79.22	74.17

**Table 2:** The overall accuracy (%) obtained in SBs of the TQWT for HOCV, FFCV, and TFCV techniques using different classification scenarios.

Class	SB-1	SB-2	SB-3	SB-4	SB-5	SB-6	SB-7	SB-8
HOCV								
D vs. F	95.22	95.59	94.12	97.06	95.51	94.12	95.59	96.32
UF vs. F	93.01	92.65	93.38	90.81	86.03	86.40	88.24	92.65
D vs. UF	90.74	92.65	93.75	88.24	91.18	84.19	84.19	84.93
D vs. UF vs. F	85.78	85.34	85.64	85.49	83.14	81.86	79.71	83.68
FFCV								
D vs. F	96.10	95.07	94.41	95.51	94.49	94.19	94.12	96.32
UF vs. F	91.32	90.59	91.69	90.96	89.49	87.57	84.85	90.96
D vs. UF	90.74	90.44	89.93	88.31	89.04	87.50	86.99	85.74
D vs. UF vs. F	89.82	87.25	86.81	86.47	85.78	83.87	81.32	85.00
TFCV								
D vs. F	94.85	95.00	94.19	95.37	95.00	94.19	93.75	95.74
UF vs. F	90.74	89.71	90.66	90.07	89.26	87.72	84.85	88.24
D vs. UF	90.22	90.00	88.53	88.16	87.87	84.85	84.93	84.26
D vs. UF vs. F	89.02	85.34	85.64	85.49	83.14	81.86	79.71	83.68

**Table 3:** The overall accuracy (%) obtained in SBs of the FAWT for HOCV, FFCV, and TFCV techniques using different classification scenarios.

Class	SB-1	SB-2	SB-3	SB-4	SB-5	SB-6	SB-7
HOCV							
D vs. F	90.44	88.82	89.29	86.40	93.01	89.13	97.79
UF vs. F	86.76	83.82	79.41	81.25	80.15	83.46	93.75
D vs. UF	83.60	81.99	81.25	83.82	84.19	80.88	92.28
D vs. UF vs. F	76.47	76.42	76.52	76.23	77.01	75.34	91.01
FFCV							
D vs. F	88.38	87.87	88.46	88.16	90.15	87.57	96.91
UF vs. F	83.38	82.72	83.60	81.84	82.43	81.25	93.09
D vs. UF	81.03	82.06	80.00	81.84	81.91	80.51	91.10
D vs. UF vs. F	73.73	76.12	75.86	76.11	76.97	75.34	90.90
TFCV							
D vs. F	87.28	87.35	88.24	86.47	88.16	86.62	96.84
UF vs. F	81.76	82.13	82.13	82.06	82.13	81.91	92.94
D vs. UF	77.79	78.90	78.31	79.19	79.63	79.26	90.96
D vs. UF vs. F	73.73	74.22	75.83	75.15	73.73	73.28	90.10

**Table 4:** The overall accuracy (%) of fused features of best performing SBs of ensemble decomposition techniques.

No. of Features	TQWT/FAWT	TQWT/MDWT	MDWT/FAWT	Fused Model
One	86.2	81.83	81.51	86.3
Two	88.26	87.7	88.24	89
Three	86.61	82.5	84.61	91.62
Four	90.98	88.62	89.61	92.45
Five	90.04	88.24	88.62	92.24
Six	90.83	88.62	89.61	91.6
IMV	–	–	–	97.8

**Table 5:** Performance parameters obtained for different scenarios of the proposed model.

Measures	Recall (%)	SPE (%)	PPV (%)	F1 Score (%)
Features	FAWT			
Drowsy	91.45	94.90	89.71	90.57
Focused	92.30	97.54	95.15	93.70
Unfocused	86.46	92.76	85.44	85.95
Features	MDWT + FAWT			
Drowsy	90.22	95.42	90.88	90.55
Focused	91.63	95.88	91.76	91.70
Unfocused	86.94	93.12	86.18	86.56
Features	MDWT + TQWT			
Drowsy	90.23	95.49	91.03	90.63
Focused	90.06	95.28	90.59	90.32
Unfocused	85.52	92.19	84.26	84.89
Features	FAWT + TQWT			
Drowsy	92.42	94.91	89.71	91.04
Focused	94.02	97.70	95.41	94.71
Unfocused	87.10	94.13	88.38	87.74
Features	Fused model			
Drowsy	93.13	95.91	91.76	92.44
Focused	94.48	97.78	95.59	95.03
Unfocused	89.74	94.99	90.00	89.87
Features	Majority iterative voting			
Drowsy	97.12	99.63	99.26	98.18
Focused	97.10	99.26	98.53	97.81
Unfocused	97.71	97.11	94.12	95.88

**Table 6:** Comparison with existing state-of-the-art techniques using the same dataset.

Authors	Method	Classifier	Accuracy (%)
Aci et al. [29]	STFT	KNN	77.76
		ANFIS	81.55
		SVM	91.72
Zhang et al. [30]	CNN	CNN	96.4
Khare et al. [36]	RDWT	Bagged tree	91.77
Kumar et al. [37]	PSD	KNN	97.5
Rastogi and Bhateja [38]	SWT	–	Artifact removal
**Proposed**	**Ensemble decomposition**	**Optimizable ensemble**	**97.8**

## Data Availability

No new data have been created in this research. The dataset was taken from a public repository (https://www.kaggle.com/datasets/inancigdem/eeg-data-for-mental-attention-state-detection) (accessed on 22 May 2021).

## References

[1] Nicolas-Alonso L.F., Gomez-Gil J. (2012). Brain Computer Interfaces, a Review. Sensors.

[2] Ortiz-Echeverri C.J., Salazar-Colores S., Rodríguez-Reséndiz J., Gómez-Loenzo R.A. (2019). A New Approach for Motor Imagery Classification Based on Sorted Blind Source Separation, Continuous Wavelet Transform, and Convolutional Neural Network. Sensors.

[3] Sterman M.B. (1996). Physiological origins and functional correlates of EEG rhythmic activities: Implications for self-regulation. Biofeedback Self-Regul..

[4] Nisha A.V., Pallikonda Rajasekaran M., Priya R.K., Al Bimani A. Artificial Intelligence based Neurodegenerative Disease Diagnosis and Research Analysis using Functional MRI (FMRI): A Review. Proceedings of the 2021 3rd International Conference on Advances in Computing, Communication Control and Networking (ICAC3N).

[5] Eastmond C., Subedi A., De S., Intes X. (2022). Deep learning in fNIRS: A review. Neurophotonics.

[6] Fred A.L., Kumar S.N., Kumar Haridhas A., Ghosh S., Purushothaman Bhuvana H., Sim W.K.J., Vimalan V., Givo F.A.S., Jousmäki V., Padmanabhan P. (2022). A Brief Introduction to Magnetoencephalography (MEG) and Its Clinical Applications. Brain Sci..

[7] Rim B., Sung N.J., Min S., Hong M. (2020). Deep Learning in Physiological Signal Data: A Survey. Sensors.

[8] Neri L., Oberdier M.T., van Abeelen K.C.J., Menghini L., Tumarkin E., Tripathi H., Jaipalli S., Orro A., Paolocci N., Gallelli I. (2023). Electrocardiogram Monitoring Wearable Devices and Artificial-Intelligence-Enabled Diagnostic Capabilities: A Review. Sensors.

[9] Markiewicz R., Markiewicz-Gospodarek A., Dobrowolska B. (2022). Galvanic Skin Response Features in Psychiatry and Mental Disorders: A Narrative Review. Int. J. Environ. Res. Public Health.

[10] Aricò P., Borghini G., Di Flumeri G., Sciaraffa N., Colosimo A., Babiloni F. (2017). Passive BCI in Operational Environments: Insights, Recent Advances, and Future Trends. IEEE Trans. Biomed. Eng..

[11] Borghini G., Astolfi L., Vecchiato G., Mattia D., Babiloni F. (2014). Measuring neurophysiological signals in aircraft pilots and car drivers for the assessment of mental workload, fatigue and drowsiness. Neurosci. Biobehav. Rev..

[12] Aricò P., Borghini G., Di Flumeri G., Colosimo A., Pozzi S., Babiloni F., Coyle D. (2016). Chapter 10—A passive brain–computer interface application for the mental workload assessment on professional air traffic controllers during realistic air traffic control tasks. Brain-Computer Interfaces: Lab Experiments to Real-World Applications.

[13] Khare S., Nishad A., Upadhyay A., Bajaj V. (2020). Classification of emotions from EEG signals using time-order representation based on the S-transform and convolutional neural network. Electron. Lett..

[14] Khare S.K., Bajaj V. (2020). Entropy based Drowsiness Detection using Adaptive Variational Mode Decomposition. IEEE Sens. J..

[15] Khare S.K., Bajaj V., Acharya U.R. (2023). SchizoNET: A robust and accurate Margenau–Hill time-frequency distribution based deep neural network model for schizophrenia detection using EEG signals. Physiol. Meas..

[16] Yin Y., Cai X., Ouyang M., Li S., Li X., Wang P. (2023). FoMO and the brain: Loneliness and problematic social networking site use mediate the association between the topology of the resting-state EEG brain network and fear of missing out. Comput. Hum. Behav..

[17] Örün Ö., Akbulut Y. (2019). Effect of multitasking, physical environment and electroencephalography use on cognitive load and retention. Comput. Hum. Behav..

[18] Shahabi H., Moghimi S. (2016). Toward automatic detection of brain responses to emotional music through analysis of EEG effective connectivity. Comput. Hum. Behav..

[19] Myrden A., Chau T. (2017). A Passive EEG-BCI for Single-Trial Detection of Changes in Mental State. IEEE Trans. Neural Syst. Rehabil. Eng..

[20] Li Y., Li X., Ratcliffe M., Liu L., Qi Y., Liu Q. A Real-Time EEG-Based BCI System for Attention Recognition in Ubiquitous Environment. Proceedings of the 2011 International Workshop on Ubiquitous Affective Awareness and Intelligent Interaction (UAAII ’11).

[21] Nuamah J., Seong Y. (2017). Support vector machine (SVM) classification of cognitive tasks based on electroencephalography (EEG) engagement index. Brain-Comput. Interfaces.

[22] Liu N.H., Chiang C.Y., Chu H.C. (2013). Recognizing the Degree of Human Attention Using EEG Signals from Mobile Sensors. Sensors.

[23] Ke Y., Long C., Fu L., Jia Y., Li P., Qi H., Zhou P., Zhang L., Wan B., Ming D. (2013). Visual Attention Recognition Based on Nonlinear Dynamical Parameters of EEG. Bio-Med. Mater. Eng..

[24] Wang Y., Jung T., Lin C. (2015). EEG-Based Attention Tracking During Distracted Driving. IEEE Trans. Neural Syst. Rehabil. Eng..

[25] Djamal E.C., Pangestu D.P., Dewi D.A. EEG-based recognition of attention state using wavelet and support vector machine. Proceedings of the 2016 International Seminar on Intelligent Technology and Its Applications (ISITIA).

[26] Hamadicharef B., Zhang H., Guan C., Wang C., Phua K.S., Tee K.P., Ang K.K. Learning EEG-based spectral-spatial patterns for attention level measurement. Proceedings of the 2009 IEEE International Symposium on Circuits and Systems.

[27] Mardi Z., Ashtiani S., Mikaeili M. (2011). EEG-based Drowsiness Detection for Safe Driving Using Chaotic Features and Statistical Tests. J. Med. Signals Sens..

[28] Richer R., Zhao N., Amores J., Eskofier B.M., Paradiso J.A. Real-time Mental State Recognition using a Wearable EEG. Proceedings of the 2018 40th Annual International Conference of the IEEE Engineering in Medicine and Biology Society (EMBC).

[29] Acı Ç.İ., Kaya M., Mishchenko Y. (2019). Distinguishing mental attention states of humans via an EEG-based passive BCI using machine learning methods. Expert Syst. Appl..

[30] Zhang D., Cao D., Chen H. Deep Learning Decoding of Mental State in Non-Invasive Brain Computer Interface. Proceedings of the International Conference on Artificial Intelligence, Information Processing and Cloud Computing.

[31] Islam M., Lee T. Multivariate Empirical Mode Decomposition of EEG for Mental State Detection at Localized Brain Lobes. Proceedings of the 2022 44th Annual International Conference of the IEEE Engineering in Medicine & Biology Society (EMBC).

[32] Tiwari A., Arora A., Goel V., Khemchandani V., Chandra S., Pandey V. A Deep Learning Approach to Detect Sustained Attention in Real-Time Using EEG Signals. Proceedings of the 2021 International Conference on Computational Performance Evaluation (ComPE).

[33] Samima S., Sarma M. (2022). Mental workload level assessment based on compounded hysteresis effect. Cogn. Neurodyn..

[34] Mohdiwale S., Sahu M., Sinha G.R., Bajaj V. (2020). Automated Cognitive Workload Assessment Using Logical Teaching Learning-Based Optimization and PROMETHEE Multi-Criteria Decision Making Approach. IEEE Sens. J..

[35] Easttom C., Alsmadi I. A Comparitive Study of Machine Learning Algorithms for Identifying Mental States from EEG Recordings. Proceedings of the 2022 IEEE 12th Annual Computing and Communication Workshop and Conference (CCWC).

[36] Khare S.K., Bajaj V., Sengur A., Sinha G. (2022). Classification of mental states from rational dilation wavelet transform and bagged tree classifier using EEG signals. Artificial Intelligence-Based Brain-Computer Interface.

[37] Kumar R.S., Srinivas K.K., Peddi A., Vardhini P.A.H. Artificial Intelligence based Human Attention Detection through Brain Computer Interface for Health Care Monitoring. Proceedings of the 2021 IEEE International Conference on Biomedical Engineering, Computer and Information Technology for Health (BECITHCON).

[38] Rastogi A., Bhateja V. (2021). Pre-processing of electroencephalography signals using stationary wavelet transform-enhanced fixed-point fast-ICA. Proceedings of the Data Engineering and Intelligent Computing: Proceedings of ICICC 2020.

[39] https://www.kaggle.com/inancigdem/eeg-data-for-mental-attention-state-detection.

[40] Parkale Y.V., Nalbalwar S.L. (2016). Application of 1-D discrete wavelet transform based compressed sensing matrices for speech compression. SpringerPlus.

[41] Selesnick I.W. (2011). Wavelet transform with tunable Q-factor. IEEE Trans. Signal Process..

[42] Bayram I. (2012). An analytic wavelet transform with a flexible time-frequency covering. IEEE Trans. Signal Process..

[43] Sharma M., Pachori R.B., Rajendra Acharya U. (2017). A new approach to characterize epileptic seizures using analytic time-frequency flexible wavelet transform and fractal dimension. Pattern Recognit. Lett..

[44] Khare S.K., March S., Barua P.D., Gadre V.M., Acharya U.R. (2023). Application of data fusion for automated detection of children with developmental and mental disorders: A systematic review of the last decade. Inf. Fusion.

[45] Yaacob H., Hossain F., Shari S., Khare S.K., Ooi C.P., Acharya U.R. (2023). Application of Artificial Intelligence Techniques for Brain–Computer Interface in Mental Fatigue Detection: A Systematic Review (2011–2022). IEEE Access.

[46] Flood M.W., Grimm B. (2021). EntropyHub: An open-source toolkit for entropic time series analysis. PLoS ONE.

[47] Too J., Abdullah A.R., Mohd Saad N., Tee W. (2019). EMG feature selection and classification using a Pbest-guide binary particle swarm optimization. Computation.

[48] Too J., Abdullah A.R., Saad N.M. (2019). Classification of hand movements based on discrete wavelet transform and enhanced feature extraction. Int. J. Adv. Comput. Sci. Appl..

[49] Sudarshan V.K., Acharya U.R., Oh S.L., Adam M., Tan J.H., Chua C.K., Chua K.P., San Tan R. (2017). Automated diagnosis of congestive heart failure using dual tree complex wavelet transform and statistical features extracted from 2 s of ECG signals. Comput. Biol. Med..

[50] Baygin M., Barua P.D., Dogan S., Tuncer T., Key S., Acharya U.R., Cheong K.H. (2022). A hand-modeled feature extraction-based learning network to detect grasps using sEMG signal. Sensors.

[51] Thornton C., Hutter F., Hoos H.H., Leyton-Brown K. Auto-WEKA: Combined selection and hyperparameter optimization of classification algorithms. Proceedings of the 19th ACM SIGKDD International Conference on Knowledge Discovery and Data Mining.

[52] Bühlmann P., Gentle J.E., Härdle W.K., Mori Y. (2012). Bagging, Boosting and Ensemble Methods. Handbook of Computational Statistics: Concepts and Methods.

[53] Luque A., Carrasco A., Martín A., de las Heras A. (2019). The impact of class imbalance in classification performance metrics based on the binary confusion matrix. Pattern Recognit..

[54] Baygin M., Barua P.D., Chakraborty S., Tuncer I., Dogan S., Palmer E.E., Tuncer T., Kamath A.P., Ciaccio E.J., Acharya U.R. (2023). CCPNet136: Automated detection of schizophrenia using carbon chain pattern and iterative TQWT technique with EEG signals. Physiol. Meas..

